# 保留迷走神经肺支对I期周围型肺腺癌患者术后咳嗽影响的初步研究

**DOI:** 10.3779/j.issn.1009-3419.2023.102.48

**Published:** 2024-02-20

**Authors:** Gaoxiang WANG, Zhengwei CHEN, Mingsheng WU, Tian LI, Xiaohui SUN, Meiqing XU, Mingran XIE

**Affiliations:** 230001 合肥，中国科学技术大学附属第一医院胸外科; Department of Thoracic Surgery, The First Affiliated Hospital of University of Science and Technology of China, Hefei 230001, China

**Keywords:** 肺肿瘤, 迷走神经, 咳嗽, 中文版莱斯特咳嗽量表, Lung neoplasms, Nervi vagus, Cough, Mandarin Chinese version of The Leicester Cough Questionnaire

## Abstract

**背景与目的** 咳嗽是肺部手术后的主要并发症之一，严重影响患者术后生活质量。保留迷走神经肺支可能降低患者术后咳嗽发生率。因此，本研究旨在探究保留迷走神经肺支是否能够降低I期肺腺癌患者术后慢性咳嗽发生率。**方法** 前瞻性选取2022年6月至2023年6月于中国科学技术大学附属第一医院胸外科行单孔胸腔镜肺癌根治术患者125例，根据术中是否保留迷走神经肺支分为保留迷走神经肺支组（n=61）和传统组（n=64）。记录两组患者一般临床资料、围手术期情况、淋巴结清扫情况、术前及术后8周中文版莱斯特咳嗽问卷（Mandarin Chinese version of the Leicester Cough Questionnaire, LCQ-MC）评分。将两组患者根据术中淋巴结清扫后是否填塞自体脂肪或吸收性明胶海绵分为填塞组及非填塞组，比较两组亚组间LCQ-MC评分及术后慢性咳嗽情况。**结果** 传统组术后8周LCQ-MC评分在生理、心理、社会及总分方面明显低于保留迷走神经肺支组，差异均有统计学意义（P<0.05）；咳嗽患者较保留迷走神经肺支组更多（P=0.006）。保留迷走神经肺支组和传统组分别进行亚组分析，保留迷走神经肺支组患者和传统组患者中，非填塞组术后8周LCQ-MC评分均低于填塞组（P<0.05），非填塞组术后8周咳嗽患者均较填塞组更多（P=0.001, P=0.024）。**结论** 对于I期周围型肺腺癌患者行手术治疗时，保留迷走神经肺支安全有效，能够降低患者术后慢性咳嗽发生率，提高患者术后生活质量。

## Abstract (en)

**Background and objective** Cough is one of the main complications after pulmonary surgery, which seriously affects the postoperative quality of life. Preserving the pulmonary branch of vagus nerve may reduce the incidence of postoperative cough. Therefore, the aim of this study was to investigate whether preserving the pulmonary branch of the vagus nerve could reduce the incidence of postoperative chronic cough in patients with stage I peripheral lung adenocarcinoma. **Methods** A total of 125 patients who underwent single-port thoracoscopic radical resection for lung cancer in the Department of Thoracic Surgery, The First Affiliated Hospital of University of Science and Technology of China from June 2022 to June 2023 were retrospectively selected, and divided into two groups according to whether the vagopulmonary branch was preserved during the operation, namely, the vagopulmonary branch group (n=61) and the traditional group (n=64). The general clinical data, perioperative conditions, lymph node dissection, Mandarin Chinese version of The Leicester Cough Questionnaire (LCQ-MC) scores before and 8 weeks after operation were recorded in the two groups. Both the two groups were divided into tamponade group and non-tamponade group according to whether autologous fat or gelatin sponge was tamponade after lymph node dissection. LCQ-MC scores and postoperative chronic cough of both groups were calculated. **Results** The LCQ-MC score of the traditional group was significantly lower than that of the vagopulmonary branch group in physiological, psychological, social and total scores at 8 weeks after surgery, and the difference was statistically significant (P<0.05). There were more cough patients in the traditional group than the vagopulmonary branch group at 8 weeks after surgery, with significant difference (P=0.006). Subgroup analysis was conducted separately for the vagopulmonary branch group and the traditional group. Among the patients in the vagopulmonary branch group and the traditional group, the LCQ-MC scores of the non-tamponade group 8 weeks after surgery were lower than those of the tamponade group (P<0.05). There were more patients with cough in the group 8 weeks after surgery than in the tamponade group (P=0.001, P=0.024). **Conclusion** For patients with stage I peripheral lung adenocarcinoma, the preservation of the pulmonary branch of vagus nerve is safe and effective, which can reduce the incidence of postoperative chronic cough and improve the postoperative quality of life of the patients.

随着影像学技术的发展及低剂量螺旋计算机断层扫描（computed tomography, CT）的普及，越来越多的肺部小结节被发现，其中部分患者需要行手术治疗。术后咳嗽是肺部手术患者的主要并发症之一，严重影响患者术后生活质量^[1⇓-3]^。近年来，肺部手术后咳嗽已经成为胸外科医生研究的热点问题之一。目前，已有研究^[4⇓-6]^表明手术方式、淋巴结清扫、麻醉时间及麻醉药物等因素可造成患者术后咳嗽的发生。淋巴结清扫过程中损伤迷走神经肺支可能会造成患者术后咳嗽，但缺乏有力的证据。随着手术技术与腔镜设备的进步，保留神经已经成为精准医疗的新要求。但是在肺癌手术中对迷走神经肺支的保留一直存在争议。因此，本研究使用莱斯特咳嗽问卷中文版（Mandarin Chinese version of the Leicester Cough Questionnaire, LCQ-MC）前瞻性分析胸腔镜下保留迷走神经肺支肺癌根治术对I期周围型肺腺癌患者术后咳嗽的影响。

## 1 资料与方法

### 1.1 一般资料

前瞻性选取2022年6月至2023年6月于中国科学技术大学附属第一医院胸外科行单孔胸腔镜肺癌根治术患者125例，其中男性48例，女性77例，平均年龄为（57.89±9.24）岁。纳入标准：（1）行单孔胸腔镜肺癌根治术；（2）周围型肺癌且术后病理诊断为I期肺腺癌；（3）无迷走神经侵犯；（4）术前无呼吸道感染性疾病、咽炎、过敏性鼻炎、慢性阻塞性肺疾病（chronic obstructive pulmonary disease, COPD）、支气管哮喘、鼻后滴流综合征等。排除标准：（1）中转开胸手术；（2）中央型肺癌或接受袖式切除或气管重建手术；（3）术后出现严重并发症，包括严重肺部感染、肺栓塞、乳糜胸等；（4）失访或病例资料不完整。本研究经中国科学技术大学附属第一医院伦理审查委员会批准，患者均签署知情同意书。

根据术中是否保留迷走神经肺支分为保留迷走神经肺支组（n=61）和传统组（n=64）。统计围手术期指标、术前及术后8周LCQ-MC评分情况。所有手术均由同一手术医师团队完成。数据收集及问卷调查医师对患者分组情况不知情。

患者术前常规检查包括：血常规、生化、凝血象、免疫组合，心电图、超声心动图，肺功能。同时行胸部平扫+增强CT明确病变性质。肿瘤直径>2 cm时行腹部+双侧肾上腺彩超、头颅磁共振成像平扫+增强、骨扫描检查以排除远处转移。必要时行正电子发射计算机断层显像（positron emission tomography/CT, PET/CT）检查。所有患者的肿瘤分期采用第8版肿瘤原发灶-淋巴结-转移（tumor-node-metastasis, TNM）分期系统。

### 1.2 手术方法

所有患者均行单孔胸腔镜肺癌根治术，手术方法基本一致^[7]^。两组唯一区别为保留迷走神经肺支组在术中保留迷走神经肺支，而传统组术中切断迷走神经肺支。在清扫第7组隆突下淋巴结时，分离组织时遇到的迷走神经肺支予以保留，避免其损伤或离断（图1）。部分患者淋巴结清扫后填充自体脂肪或吸收性明胶海绵（图2）。

**图1:**
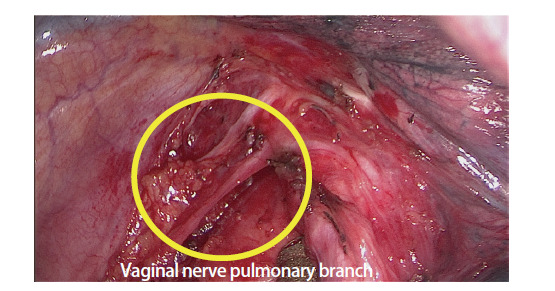
迷走神经肺支

**图2:**
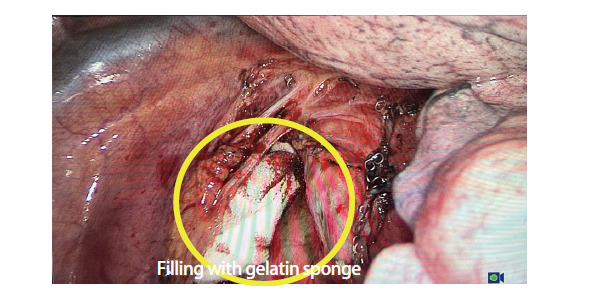
填塞明胶海绵

### 1.3 观察指标

记录两组患者性别、年龄、吸烟史、术前合并症、肿瘤部位、肿瘤最大径、麻醉时间、麻醉方式、术后带管时间、胸引管引流量、术后住院时间、淋巴结清扫站数、淋巴结清扫枚数及第7组淋巴结数目、术后并发症及术后是否服用止咳药物。术前及术后8周进行LCQ-MC评分。

通过电话随访或门诊复查调查问卷的方式比较两组患者术后慢性咳嗽情况及生活质量情况。本研究采用数字评分法（numeric rating scale, NRS）评价患者的咳嗽程度，0分为无咳嗽；1-3分为轻度咳嗽，对睡眠无影响；4-6分为中度咳嗽，对睡眠有影响，但能入睡；7-9分为重度咳嗽，无法入睡或睡眠中咳醒；10分为剧烈咳嗽。LCQ-MC评估患者咳嗽严重程度对生活质量的影响，LCQ-MC分为生理、心理和社会三个维度，共19道题，包括8项生理项目、7项心理项目和4项社会项目，每道题7个选项（正向计分，1-7个等级，分数越高表示咳嗽程度越轻）。各维度的得分由各维度题目分值取平均值（1-7分），总分为3个维度得分之和（3-21分）。术前及术后LCQ-MC克朗巴赫α系数分别为0.710及0.936。由2名经过培训的医务人员分别于术前1天及术后8周完成LCQ-MC评分。

### 1.4 统计学方法

采用SPSS 26.0 统计学软件对数据进行分析。检验数据的正态性，正态分布资料用均数±标准差（Mean±SD）表示，计量资料比较采用t检验；偏态资料以中位数（P25, P75）描述，两组间比较为Wilcoxon秩和检验。计数资料比较采用χ^2^检验。P<0.05为差异有统计学意义。

## 2 结果

### 2.1 两组患者临床资料情况比较

两组患者在性别、年龄、吸烟史、术前合并症、肿瘤部位、肿瘤最大径及麻醉方式方面的差异均无统计学意义（P>0.05）（表1）。

**表1:** 两组患者一般资料对比

Item	Vagopulmonary branch group (n=61)	Traditional group (n=64)	χ^2^/t	P
Gender			0.024	0.876
Male	23 (37.70%)	25 (39.06%)		
Female	38 (62.30%)	39 (60.94%)		
Age (yr)			0.001	0.974
<60	37 (60.66%)	39 (60.94%)		
≥60	24 (39.34%)	25 (39.06%)		
Smoking			2.178	0.140
Yes	10 (16.39%)	5 (7.81%)		
No	51 (83.61%)	59 (92.19%)		
Preoperative comorbidity			0.140	0.709
Yes	20 (32.79%)	19 (29.69%)		
No	41 (67.21%)	45 (70.31%)		
Tumor location			2.541	0.637
RUL	22 (36.07%)	18 (28.12%)		
RML	7 (11.48%)	13 (20.31%)		
RLL	12 (19.67%)	10 (15.63%)		
LUL	10 (16.39%)	12 (18.75%)		
LLL	10 (16.39%)	11 (17.19%)		
Tumor diameter (mm)	16.41±5.89	17.81±6.12	-1.305	0.194
Mode of anesthesia			1.901	0.168
Double-lumen tube	46 (75.41%)	41 (64.06%)		
Laryngeal mask	15 (24.59%)	23 (35.94%)		

RUL: right upper lung; RML: right middle lung; RLL: right lower lung; LUL: left upper lung; LLL: left lower lung.

### 2.2 两组患者围手术期情况比较

两组患者在麻醉时间、术后带管时间、胸引管引流量、术后住院时间、淋巴结清扫站数、淋巴结清扫数目、第7组淋巴结清扫数目及术后并发症方面的差异均无统计学意义（P>0.05）。传统组患者术后使用止咳药物占比较保留迷走神经肺支组更多，差异有统计学意义（P=0.013）（表2）。

**表2:** 两组患者围手术期情况比较

Item	Vagopulmonary branch group (n=61)	Traditional group (n=64)	t/Z/χ^2^	P
Anesthesia time (min)	141.52±38.63	155.50±48.41	-1.779	0.078
Chest tube duration (d)	4 (4, 4)	4 (3, 6)	-1.025	0.306
Postoperative hospital stay (d)	5 (4, 5)	5 (4, 7)	-0.634	0.526
Postoperative thoracic drainage (mL)	535.0 (355.0, 570.0)	447.5 (345.0, 582.5)	-0.786	0.432
The stations of the total lymph nodes dissected	6 (6, 6)	6 (6, 6)	-0.469	0.639
The number of the total lymph nodes dissected	12 (10, 13)	12 (10, 14)	-0.701	0.483
The number of lymph node dissection in group 7	3 (2, 5)	4 (2, 5)	-0.274	0.784
Postoperative complications			0.858	0.354
Yes	19 (31.15%)	25 (39.06%)		
No	42 (68.85%)	39 (60.94%)		
Postoperative cough medication			6.140	0.013
Yes	10 (16.39%)	23 (35.94%)		
No	51 (83.61%)	41 (64.06%)		

Postoperative complications include pulmonary inflammation, atelectasis, pleural effusion, etc.

### 2.3 两组LCQ-MC评分比较

术前两组LCQ-MC评分在生理、心理、社会及总分方面均无统计学差异（P>0.05）。术后8周传统组LCQ-MC评分在生理、心理、社会及总分方面明显低于保留迷走神经肺支组，差异均有统计学意义（P<0.05）。传统组术后8周咳嗽患者较保留迷走神经肺支组更多，差异有统计学意义（P=0.006）（表3）。

**表3:** 两组患者LCQ-MC评分比较及术后慢性咳嗽情况对比

Item		Vagopulmonary branch group (n=61)	Traditional group (n=64)	t/χ^2^	P
Physical	Preoperative	6.71±0.18	6.68±0.19	0.988	0.325
	8 weeks after surgery	6.26±0.52	5.77±0.68	4.592	<0.001
Psychological	Preoperative	6.63±0.18	6.60±0.24	0.784	0.437
	8 weeks after surgery	6.20±0.50	5.73±0.60	4.793	<0.001
Social	Preoperative	6.71±0.18	6.72±0.20	-0.063	0.950
	8 weeks after surgery	6.15±0.54	5.60±0.76	4.662	<0.001
Total scores	Preoperative	20.05±0.33	19.99±0.42	0.877	0.382
	8 weeks after surgery	18.61±1.49	17.10±1.94	4.920	<0.001
Postoperative chronic cough	Yes	15 (24.59%)	31 (48.43%)	7.637	0.006
	No	46 (75.41%)	33 (51.56%)		

LCQ-MC: Mandarin Chinese version of the Leicester Cough Questionnaire.

### 2.4 亚组分析

将保留迷走神经肺支组患者及传统组患者根据术中淋巴结清扫后是否填塞自体脂肪或吸收性明胶海绵分为填塞组及非填塞组。

保留迷走神经肺支组亚组分组情况为填塞组38例，非填塞组23例。两组术前LCQ-MC评分在生理、心理、社会及总分方面均无统计学差异（P>0.05）。术后8周LCQ-MC评分在生理、心理、社会及总分方面，非填塞组均明显低于填塞组（P<0.05）。保留迷走神经肺支组中非填塞组术后8周咳嗽患者较填塞组患者更多，差异有统计学意义（P=0.001）（表4）。

**表4:** 保留迷走神经肺支组淋巴结清扫后填充与不填充患者LCQ-MC评分对比及术后慢性咳嗽情况对比

Item		Tamponade group (n=38)	Non-tamponade group (n=23)	t/χ^2^	P
Physical	Preoperative	6.70±0.17	6.72±0.19	-0.356	0.723
	8 weeks after surgery	6.42±0.40	6.00±0.60	3.001	0.005
Psychological	Preoperative	6.63±0.17	6.62±0.21	0.263	0.793
	8 weeks after surgery	6.34±0.37	5.98±0.60	2.603	0.014
Social	Preoperative	6.70±0.17	6.74±0.18	-0.938	0.352
	8 weeks after surgery	6.34±0.37	5.84±0.64	3.399	0.002
Total scores	Preoperative	20.03±0.31	20.08±0.37	-0.538	0.592
	8 weeks after surgery	19.10±1.04	17.81±1.77	3.166	0.003
Postoperative chronic cough	Yes	4 (10.53%)	11 (47.83%)	10.750	0.001
	No	34 (89.47%)	12 (52.17%)		

传统组亚组分组情况为填塞组40例，非填塞组24例。两组术前LCQ-MC评分在生理、心理、社会及总分方面均无统计学差异（P>0.05）。术后8周LCQ-MC评分在生理、心理、社会及总分方面，非填塞组均明显低于填塞组，差异均有统计学意义（P<0.05）。传统组中非填塞组术后8周咳嗽患者较填塞组患者更多，差异有统计学意义（P=0.024）（表5）。

**表5:** 传统组淋巴结清扫后填充与不填充患者LCQ-MC评分对比及术后慢性咳嗽情况对比

Item		Filled group (n=40)	Unfilled group (n=24)	t/χ^2^	P
Physical	Preoperative	6.65±0.20	6.72±0.15	-1.553	0.125
	8 weeks after surgery	5.91±0.65	5.53±0.68	2.171	0.034
Psychological	Preoperative	6.60±0.24	6.60±0.22	-0.019	0.985
	8 weeks after surgery	5.86±0.56	5.51±0.62	2.295	0.025
Social	Preoperative	6.71±0.29	6.73±0.20	-0.516	0.607
	8 weeks after surgery	5.82±0.59	5.23±0.86	3.230	0.002
Total scores	Preoperative	19.95±0.44	20.06±0.40	-0.929	0.356
	8 weeks after surgery	17.58±1.73	16.28±2.04	2.733	0.008
Postoperative chronic cough	Yes	15 (37.50%)	16 (66.67%)	5.109	0.024
	No	25 (62.50%)	8 (33.33%)		

## 3 讨论

咳嗽是肺部手术后常见并发症之一，控制不佳将转变为慢性咳嗽，严重影响患者术后生活质量。引起肺部术后咳嗽的原因多样，其中术中切断迷走神经肺支可能是增加患者术后咳嗽风险的因素之一。迷走神经在呼吸系统中扮演重要角色。已有研究^[8,9]^证实，迷走神经肺支在咳嗽反射、排痰及肺炎免疫防御中起到重要作用。本研究发现，保留迷走神经肺支能够降低患者术后慢性咳嗽的发生率，其术后8周LCQ-MC评分均优于传统组患者。

本研究中保留迷走神经肺支组与传统组在手术方式上基本一致。两组患者围手术期指标及术后并发症方面均无明显统计学差异，说明保留迷走神经肺支在手术治疗效果方面安全有效。保留迷走神经肺支患者术后止咳药物使用情况较不保留迷走神经肺支的患者更少，说明不保留迷走神经肺支的患者术后出现咳嗽的概率更高，术后生活质量更低。另外，保留迷走神经肺支使患者神经得到了保留，患者受到的创伤更小，从某种方面而言间接保留了患者的某种生理功能，使患者受益，提高患者术后生活质量。

LCQ-MC被证实有很好的可靠性、可重复性和灵敏度，使用其评估肺部手术后患者的咳嗽情况效果良好^[10]^。本研究发现，保留迷走神经肺支的患者术后8周LCQ-MC评分各方面均优于不保留迷走神经肺支的患者，且保留迷走神经肺支的患者术后慢性咳嗽发生率更低。Gu等^[11]^通过一项纳入158例行胸腔镜肺癌根治术的非小细胞肺癌患者的随机双盲试验发现，保留肺迷走神经分支的患者术后2和5周LCQ-MC评分显著高于常规手术患者，保留肺迷走神经分支可降低术后咳嗽的发生率。张楠等^[12]^通过一项纳入120例行胸腔镜上叶肺癌根治术的非小细胞肺癌患者的前瞻性随机对照试验发现，保留迷走神经组患者LCQ-MC评分各方面均优于传统组，咳嗽情况有所减轻，胸腔镜上叶肺癌根治术中保留迷走神经肺支可减轻术后咳嗽的发生，对于患者加速康复具有重要作用。保留迷走神经肺支能够降低患者术后咳嗽的机制可能有以下几种：第一，由于手术最大化地保留了迷走神经的完整性，使余肺的生理功能及神经通路更加接近术前，从而降低患者术后咳嗽的发生率。第二，迷走神经在咳嗽中起重要作用。瞬时感受器电位香草酸亚型1（transient receptor potential vanilloid subfamily 1, TRPV1）受体在迷走神经C纤维上表达并参与咳嗽调控，术中迷走神经肺支损伤可促进缓激肽和前列腺素E2（prostaglandin E2, PGE2）等炎症因子的释放，从而激活TRPV1通路并向神经中枢发送信号，使患者术后发生持续性咳嗽^[13]^。第三，迷走神经肺支损伤可能影响患者的迷走神经食管支，增加患者胃-食管反流的发生率，从而引起患者术后咳嗽。

手术引起的局部胸膜炎及神经的局部炎症同样能够诱发患者术后咳嗽。研究^[14]^发现肺癌手术中淋巴结清扫后会形成残腔，特别是在清扫隆突下淋巴结和上纵隔淋巴结清扫留下的残腔是诱发术后咳嗽的因素之一。本研究亚组分析结果显示，保留迷走神经肺支组及传统组中淋巴结清扫后填塞自体脂肪组织或吸收性明胶海绵的患者术后8周LCQ-MC评分各方面均优于非填塞的患者，填塞患者术后慢性咳嗽发生率低于非填塞组。黄佳等^[14]^通过对比30例使用前纵隔脂肪填塞上纵隔淋巴结清扫遗留的残腔的右肺癌患者和30例未使用前纵隔脂肪填塞上纵隔淋巴结清扫遗留的残腔的右肺癌患者的随机试验发现，前纵隔脂肪填塞残腔能有效减少术后顽固性咳嗽的发生，提高患者的生存质量。Wu等^[15]^研究发现，淋巴结清扫术较淋巴结采样术后咳嗽发生率更高，也从侧面说明淋巴结残腔更大可能影响患者术后咳嗽发生。填塞淋巴结残腔术后咳嗽发生率更低的机制主要有以下几种可能：第一，填塞淋巴结清扫后残腔避免术后包裹性积液形成，降低患者术后胸腔内炎症的发生，从而降低术后咳嗽发生率。第二，填塞残腔能够避免迷走神经肺支及咳嗽相关感受器暴露在外，降低外界对其刺激，从而降低患者术后咳嗽。

综上所述，对于I期周围型肺腺癌患者行手术治疗时，淋巴结清扫过程中应避免迷走神经肺支的损伤，纵隔淋巴结清扫后遗留的残腔应使用自体脂肪组织或吸收性明胶海绵填塞，避免患者术后出现慢性咳嗽，影响患者术后生活质量。但对于患者术后使用止咳药物的时间及时长没有做具体分析，可能会对结果产生一定偏倚。另外，本研究是单中心小样本研究，存在一定的选择偏倚，其结果有待大样本多中心的随机对照研究进一步证实。

**Competing interests**

The authors declare that they have no competing interests.

**Author contributions**

Wang GX, Xu MQ and Xie MR conceived and designed the study. Chen ZW and Li T performed the data collection and follow-up. Wu MS and Sun XH analyzed the data. Chen ZW contributed analysis tools. Xu MQ and Xie MR provided critical inputs on design, analysis, and interpretation of the study. All the authors had access to the data. All authors read and approved the final manuscript as submitted.
